# Report of a Case of Mercurial Stomatitis, with Remarks

**Published:** 1856-04

**Authors:** R. Bruce Reynolds


### ARTICLE VIII.

*Report of a Case of Mercurial Stomatitis, with Remarks.* By R. Bruce Reynolds, D. D. S.

Of late years, since the reckless employment of mercury, formerly so common, has been generally abandoned, the opportunities for witnessing severe cases of salivation have become fewer. As it has fallen to my lot to see one in which the local inflammation was great, the fever high, and the convalescence unusually rapid, in consequence of the prompt and satisfactory action of the remedies employed, I have thought that it was of sufficient interest to be reported in full.

Miss F——, aged about 18 years, a seamstress, of lax fibre, lymphatic temperament and scrofulous appearance, sent in haste for my friend, Dr. James Dwinelle; the messenger saying that "she was bleeding to death." I accompanied him, witnessed the entire progress of the case, and kept minutes of the various changes which took place from day to day.

On arriving we found her pallid, gasping, bleeding profusely, and apparently on the verge of syncope from exhaustion by hemorrhage. A wash bowl near her, contained fully a pint of dark, grumous blood, mixed of course with saliva, and the attendants informed us that she had lost much more than that. The mouth could only be opened about half an inch for inspection, but that was sufficient to reveal the source of the hemorrhage. Just behind the incisor teeth, in the neighorhood of the ducts of the sublingual gland, there was a fungous growth, rising as high as the level of the incisor teeth, which was at first mistaken for a clot of blood. It was soon found, however, to be very sensitive and to bleed at the slightest touch. Indeed, pressure upon it caused the blood to flow from it as if oozing from a compressed sponge. For the rest, all the salivary glands, the tongue, cheeks and gums were highly inflamed, the mucous surfaces being of a dark crimson color and a spongy texture.

The cheeks were tumid, pale and doughy, the parotids swollen and extremely tender, the complexion pale, and the fetor of the breath intolerable, clearly indicating a mercurial taint. The tongue was furred, fissured and swollen, the tonsils enlarged, and indeed the entire buccal apparatus greatly engorged. The surface was hot, the condition evidently feverish, the pulse 100 in the minute, vacuous and quick.

Upon inquiring, it was ascertained that about three weeks before she had been affected with piles, for which a neighboring practitioner had recommended mercurial ointment to the tumors, and internally confection of senna, with which it was afterwards discovered that mercury had been mixed. Both had been used several times every day, up to the day before the hemorrhage occurred.

Brandy was at once administered locally as an astringent, and internally as a stimulant.

The ulceration of the mouth being considerable, it was determined to employ nitrate of silver. A strong solution of the fused salt, (half a drachm to the fluid ounce, of distilled water,) was applied by a soft camel's-hair pencil to the bleeding surface and to all the ulcerated tissues of the mouth.

It speedily and completely arrested the hemorrhage and exerted a decided antiphlogistic influence upon the local inflammation. Solution of acetate of lead, with laudanum, was then used, as a wash, for the double purpose of constringing the vessels and soothing the excessive pain. For the purpose of correcting fetor she used interchangeably with this wash, Labarraque's solution of chlorinated soda, as a lotion. To correct the condition of the blood, chlorate of potash was administered internally by the following formula:

| | | |
|---|---|---|
| ℞ | Potassæ chloratis, . . | ℨ ss. |
| | Syrupi simplicis, . . . | ℥ ijss. |
| | Aquæ, . . . . . . | ℥ xijss. M. |

Dose, a table-spoonful three times a day.

At night, on account of the excessive irritation, she took twenty-five drops of laudanum.

Nov. 30th, 10, A. M. The patient had passed rather a restless night, her condition, however, was better, her pulse 90 and fuller, her skin soft, and the inflammation of the mouth decidedly diminished. The mouth could be opened wider so as to show small ulcers on the sides of the tongue and the mucous lining of the cheek. The nitrate of silver was again applied as before, and the same local and general treatment continued.

8, P. M. No particular change, except that she could open her mouth better.

Dec. 1st, 8 A. M. Has had a restless night, but is more comfortable—continue treatment.

7, P. M. Tongue furred; mercurial fetor gone; pulse 94. She complains of pain in the left tonsil. Her bowels being torpid, half an ounce of castor oil was given at once, and at bed time she took ten grains of Dover's powder.

Dec. 2d. She has had a good night's rest; and her bowels have been freely moved. She is in every respect improved.

Dec. 3d. She has a slight hemorrhage, but, in other respects, continues to improve. Nitrate of silver is again applied and arrests the bleeding. The other treatment is continued.

Dec. 4th. Has had a comfortable night. The tongue is clean; the mercurial odor entirely absent; the pulse 75. She has entirely recovered the use of her jaws.

8, P. M. Complains of great soreness near the root of the tongue on the gum of the left side, and upon examination, a small red and tender spot, about the size of a half dime, is discovered. Nitrate of silver is applied and the washes continued as before. From this time her convalescence was rapid. It should be remarked that during the whole course of the treatment she took wine whey, beef tea and similar sustaining diet.

*Remarks.*—It is well known that the flow of saliva may be increased by a great variety of causes, both local and general. Of the local stimuli and of the influence of the mind upon this secretion, it is not necessary to speak. The agents, which taken internally, are capable of producing this effect upon these glands, are said to be numerous. Antimony, nitric acid, iodine, chlorine, bromine, arsenic, lead and gold, among inorganic: and opium, conium, belladonna, xanthoxylum and others, among organic substances, have been accused of causing ptyalism.

Iodine is the only one of these of which I intend speaking. The following sketch of its action is taken from Piggot's Dental Chemistry.

"Iodine stands next to mercury as a general sialagogue. The mercurial fetor has been stated by some observers to be present in this form of salivation, while others deny that there is either fetor, sponginess of the gums, or loosening of the teeth. The explanation of this discrepancy will probably be found in the facts recently elicited by M. Melsens' researches. This accurate and careful observer shows that mercury may remain a very long time in the system, in consequence, probably, of its property of forming insoluble compounds with the various organic substances of which the body is composed; and that iodide of potassium is capable of dislodging it, and making it soluble, so that it will again enter the current of the blood, and may, consequently, if set free in sufficient quantity, produce its primary effects of salivation, erythema, &c. Dr. Budd relates a case in which, five months after taking mercury, a patient was violently mercurialized in this way by iodide of potassium.

"The tendency of iodine and its compounds to pass off through the salivary secretions has already been mentioned. Like most other substances, however, it may change the organ of elimination, and may be discharged through the skin, the kidneys, or even by means of the fluid evacuated in consequence of the operation of a seton. Under circumstances like these, its elimination by the salivary glands is of course checked, if not entirely suspended.

"Pure iodic salivation differs materially from that produced by mercury. The vascularity of the mucous membrane of the mouth and of the salivary glands may be unchanged, but oftener it is increased, the glands especially becoming tumid and tender. The secretion itself contains an unusual amount of mucus or albumen, the excess of one or the other of these elements, constituting its chief deviation from the healthy state. The unpleasant taste of iodine is often perceived in the secretion, and weight at the root of the tongue, aching of the jaws and singing in the ears are complained of. An increased flow of tears and of nasal mucus generally accompanies this form of salivation."

There are a few points in mercurial salivation on which some remarks may be needed. The chemistry of the saliva is greatly modified. It becomes thicker, more viscid, more mucous, more alkaline. It is nauseous to the taste, has a peculiar disagreeable, penetrating odor, which has been attributed to the greater quantity of Wright's ptyalin which has been found to be present.

The symptoms of the disease, I quote from Dr. Wood's work on practice of medicine.

"Among the first indications of the action of mercury are often a metallic taste in the mouth, like that of brass or copper, and some increase of the saliva. At the same time, a close examination will detect a slight redness and swelling of the gums, particularly about the necks of the lower incisors, while somewhat below their edge, a broad white line may often be observed, depending on opacity of the epithelium. The patient soon begins to feel some uneasiness, complaining of soreness when the gums are pressed, and of pain when the teeth are forcibly closed together. There is also a sense of stiffness about the jaws when the mouth is opened, and they feel as if projecting above their usual level. The flow of saliva increases, the inflammation extends, the gums and palate become obviously swollen, and the tongue covers itself with a yellowish-white or brownish fur, and is often so much enlarged as to exhibit the impression of the teeth when projected from the mouth. The throat frequently becomes sore, and the cheeks, and salivary and absorbent glands, swollen and painful. There is often severe tooth-ache or pain in the jaws. A whitish exudation along the edges of the gums is very common. The breath, which, from the beginning, and sometimes even before the appearance of any one of the symptoms mentioned, has a peculiar disagreeable odor, now becomes extremely offensive, and in bad cases almost intolerable. Ulceration often occurs, especially about the necks of the teeth, which are consequently loosened, and in the cheeks, lips and fauces. The ulcers often have their origin in a vesicular eruption, such as that already described.

(See page 501.) The whole mouth with its appendages is sometimes so much swollen that it can scarcely be opened; and the tongue so much enlarged as to project beyond the lips. The patient is now nearly or quite unable to articulate, or to masticate his food, and sometimes can scarcely swallow. A case was related by Dr. Physick, in his lectures, in which an obstinate dislocation of the jaw resulted from the enormous tumefaction of the tongue. Hemorrhage is not an unfrequent attendant upon these bad cases, and is sometimes so profuse as to be alarming. Sloughing also takes place, and portions of the jaw bone are occasionally laid bare. There is always, in the severe cases, more or less fever, which is partly symptomatic of the local affection, partly the direct effect of the mercury. Death, from the exhausting influence of the irritation, want of nourishment and hemorrhage, has occurred in numerous instances; but the patient generally recovers from the worst forms of the affection, though sometimes with a deformed mouth. The tongue and cheek have occasionally adhered at points where their ulcerated surfaces were in contact; and a surgical operation has been necessary to remove the evil."

In regard to the treatment, that portion of it which seemed specially efficacious was the local application of nitrate of silver and the internal use of chlorate of potash. The former arrested the hemorrhage and changed the condition of the mouth almost immediately. For the effects of the latter I refer to certain passages in recent journals which have been pointed out to me by a medical friend.

The *Bulletin de Therapeutique*, as quoted in the Dublin Medical Press, of Sept. 5th, 1855, contains a recommendation of this remedy, from Herpin & Blachthey: begin with a dose of half a drachm a day, and gradually increase it.

"M. Gustin, intern in pharmacy, wishing, for the sake of experiment, to submit himself to the action of chlorate of potash, took two drachms at nine o'clock in the evening. On awakening, a sort of astriction, with slight nausea, was perceived in the mouth; the gums were a little rough to the touch. Although the saliva was not sensibly lessened, it appeared to him to be more watery than usual. This observer has also proved that the chlorate of potash is in great part eliminated by the urinary secretions."

Under this treatment it is said all the symptoms disappear in four days. In corroboration of this, the Medical Times and Gazette, of Nov. 19th, 1855, records a case of Mr. Hutchinson, in which all fetor had disappeared on the third day. He gave the medicine in fifteen grain doses, thrice daily.

